# *Trypanosoma cruzi* Transmission Through Blood Samples and Derivatives: Main Routes, Control Strategies, and Recent Advancements in Blood Banks

**DOI:** 10.3390/pathogens14020133

**Published:** 2025-02-02

**Authors:** Aline Nefertiti Silva da Gama, Maria de Nazaré Correia Soeiro

**Affiliations:** Cell Biology Laboratory, Oswaldo Cruz Institute, Oswaldo Cruz Foundation, Rio de Janeiro 21040-900, RJ, Brazil; alinenefertitigama@gmail.com

**Keywords:** Chagas disease, blood banks, neglected tropical diseases

## Abstract

Neglected Tropical Diseases are a group of 25 conditions caused by diverse agents. They mostly affect people with poorer health outcomes, particularly preventable diseases. The social determinants of health influence the development and progression of these poverty diseases, with inadequate sanitation presenting chronicity, high morbidity, and economic impacts. Chagas disease, a prominent Neglected Tropical Disease caused by the intracellular pathogen *Trypanosoma cruzi*, is endemic in Latin America but is increasing as a global concern due to population migration. It is transmitted through insect vectors, congenitally, orally via contaminated food and beverage, via transfusions and organ donation, and due to laboratory accidents, among other minor relevant routes. As a silent illness, with many infected individuals remaining asymptomatic, it contributes to underdiagnosis, and delayed treatment that involves nitro derivatives is often discontinued due to side effects. Chagas disease spreads in non-endemic areas like the United States of America and Europe. Blood screening practices vary, with endemic regions implementing universal testing, while non-endemic areas rely on selective methods. Recent innovations, such as riboflavin–ultraviolet light treatment and arylimidamide compounds, represent promising alternatives to reduce transfusion transmission. This review presents an analysis of *Trypanosoma cruzi* transmission through blood and derivatives, addressing the main routes, globally implemented control strategies, and recent advancements in blood bank safety.

## 1. Introduction

Neglected Tropical Diseases comprise a group of 25 conditions caused by various agents, including viruses, bacteria, fungi, protozoa, helminths, and toxins [1]. These diseases share several common characteristics: (i) a strong association with inadequate or absent basic sanitation; (ii) the potential for transmission control through prophylaxis and treatment, often hindered by the lack of adequate public policies; (iii) high prevalence among impoverished populations, affecting the most vulnerable groups; (iv) a tendency toward chronicity due to late diagnosis and inadequate treatment; and (v) significant economic impact due to high morbidity rates, including healthcare costs and disability-related pensions [2,3]. These diseases affect over one billion people worldwide, causing approximately 200,000 deaths annually and leading to the loss of about 25 million disability-adjusted life years [4].

Chagas disease belongs to the group of Neglected Tropical Diseases and was discovered 115 years ago by Brazilian physician Carlos Chagas. Chagas disease is caused by the protozoan *Trypanosoma cruzi* and is endemic in 21 countries in Latin America, occurring from the southern United States to northern Argentina and Chile [5,6]. Currently, about 6–7 million people globally are infected, with 75 million people in the Americas at risk of contracting the disease [7]. As described by Chagas [8], transmission can occur through various routes: (i) vector borne, when the triatomine insect defecates during its blood meal, and the fecal material containing the parasite is introduced through the bite wound in the skin or mucous membranes; (ii) congenital (from mother to child) during pregnancy or delivery; (iii) orally, involving the consumption of food or beverages contaminated with infected triatomines or their feces; (iv) laboratory accidents; and (v) organ transplantation and blood transfusion [9,10,11].

Most acute infections are asymptomatic or exhibit nonspecific manifestations, characterized by a short duration, often 4–8 weeks, of displaying circulating parasites in the blood [12]. This clinical diversity contributes to many cases remaining undiagnosed or unnotified. Without appropriate treatment during the acute phase, the symptoms and parasitaemia are naturally controlled by an effective host immune response, and the individuals remain in the indeterminate form of the chronic phase, without presenting any symptoms for decades or even for their entire lives [13,14]. Nevertheless, 30 to 40% of infected people progress from the indeterminate form to the symptomatic chronic phase, presenting severe clinical manifestations, including digestive, cardiac, mixed (cardio-digestive), and neurological forms [14].

Treatment relies on two nitro derivatives, nifurtimox and benznidazole. Both cause severe side effects and require prolonged treatments, which often result in therapy discontinuation, in addition to the existence of parasites naturally resistant to both drugs [15,16]. Previously limited to the Americas, Chagas disease has become a global concern due to population mobility and urbanization. Today, cases are reported in countries such as Canada, the United States of America, and even parts of Europe, Africa, and the Western Pacific [17,18]. Over the past 40 years, the migration of *Trypanosoma cruzi*-infected individuals from endemic countries to non-endemic regions, particularly in North America and Europe, has raised concerns about blood safety across vast geographic areas [19]. Although initiatives have been implemented to mitigate the risk of transfusion-transmitted infections in non-endemic regions, there is still no standardized or universal approach, which hinders effective control of this transmission route [20]. This paper presents a review of the transmission of Chagas disease through blood and its derivatives, addressing the main infection routes, globally implemented control strategies, and recent advancements in technologies and medications to enhance blood bank safety. Finally, the impact of these initiatives on combating transfusion-transmitted infections and promoting public health in different regions of the world is discussed.

## 2. Blood and Blood Derivatives Transmission

Approximately 4.6 million Latin American migrants live in Europe, with an estimated 68,000 to 122,000 undiagnosed cases of Chagas disease. Determining the exact number of undocumented migrants is extremely challenging, although they are likely to have the highest prevalence rates of the disease [21]. The transfusional transmission of Chagas disease is widely recognized and has been the subject of studies since the first reports in Brazil in 1949 and 1952 [22,23]. While blood transmission, like the classic vectorial transmission, is under control in endemic regions, with serological testing being mandatory in some countries such as Brazil [24], this is not universally observed in non-endemic regions such as the United States of America, Canada, Asia, Australia, and Japan. Unlike Latin America, where control policies are more established, the process of globalization and increasing migration have contributed to the spread of Chagas disease to non-endemic areas, making vertical (mother to child) and transfusional transmission a global concern [20,25]. Many individuals affected by Chagas disease remain asymptomatic and, therefore, are unaware of their infection, which increases the risk of transmission, especially considering that, in some countries, blood donation is remunerated as an incentive. This situation represents a significant threat to the safety of the blood supply in non-endemic regions [26].

The first case of transfusional transmission of *Trypanosoma cruzi* in the United States of America was verified in 1987, and then in 1989 in Canada, and in the early 2000s in Europe, highlighting the urgent need to implement mitigation strategies in these areas [27]. The transmission of *Trypanosoma cruzi* through blood and its derivatives occurs when contaminated components are transfused to recipients [28,29]. All blood components, except for frozen fresh plasma derivatives, are considered potentially infectious. The parasite can sustain its viability for up to 18 days at 4 °C and up to 250 days at room temperature [30,31]. Although cryopreserved stocks may remain viable in frozen vials, there is no clear evidence of transmission through cellular components that were frozen and thawed [32].

In endemic countries, the likelihood of infection following the transfusion of a single unit of blood from an infected donor has been estimated at around 10–20% [33]. The infectivity of different blood components varies, with platelets being the most frequently associated with transfusional transmission of *Trypanosoma cruzi*. According to evidence from non-endemic regions, the transmission risk ranges from approximately 6–26% for platelets and only about 0–4% for packed red blood cells, plasma, and cryoprecipitates, since the bloodstream parasites concentrate in the same region as leucocytes and platelets after differential centrifugation [34]. Platelets are frequently involved in *Trypanosoma cruzi* transmission, particularly in immunocompromised patients or those undergoing oncological treatment [26]. The probability of transfusional transmission depends on multiple factors, such as the amount of blood transfused, the parasite strain, and the specific discrete type units, among others [35,36,37].

## 3. Control and Prevention Strategies in Blood Banks

Since the establishment of the practice of blood transfusion in 1940 in Latin America, Chagas disease has become a major risk factor, with the first case of transmission reported in 1952 in Brazil, but it has only been mandatory to perform serological testing in blood banks since 1969 [24]. Currently, screening is mandatory in all countries where the parasite circulates naturally, including the United States of America [38]. According to the Pan American Health Organization, in 2010, the prevalence of infection varied greatly between countries, being highest in Argentina, Paraguay, and Bolivia and relatively low in places such as Costa Rica, Chile, and Brazil. In the United States of America, although the prevalence is low, there are significant regional differences, with higher numbers in Florida and Texas [33]. While universal screening may not be economically feasible in settings with very low prevalence, it is ethically unacceptable for those recipients who may receive infected blood. Thus, even with advances in control, sporadic infections may still occur, highlighting the need to improve test sensitivity and continuously monitor mitigation strategies.

Thus, screening and control strategies to mitigate transfusion transmission of *Trypanosoma cruzi* vary widely among countries, reflecting their epidemiological realities and available resources. While endemic countries adopt universal screening and mandatory testing, non-endemic regions rely on targeted questionnaires and selective testing. Despite advances, challenges remain, highlighting the need for continued improvements, such as improving screening technologies and implementing more comprehensive global policies to ensure the safety of the blood supply.

### Control and Safety Strategies in Blood Donations in Endemic and Non-Endemic Countries

As described above, Brazil has been a pioneer in implementing mandatory universal screening. Serological testing in blood banks uses advanced methods such as chemiluminescent and enzyme-linked immunosorbent assay, with confirmations through indirect immunofluorescence and Western blot. Brazil has one of the most robust legislations, ensuring that all donors are tested for *Trypanosoma cruzi*. In positive cases, the products are discarded, and the donors are permanently excluded. The prevalence of infection in Brazil is relatively low compared to other Latin American countries, but population mobility requires continuous surveillance [39,40]. In Argentina, Bolivia, and Paraguay, there is still a high prevalence of *Trypanosoma cruzi* infection, which makes mandatory universal blood screening a crucial measure. Similarly to Brazil, these countries use highly sensitive serological methods. Local legislation ensures strict control of donations, mitigating the risks of transfusion transmission [20].

In the United States of America, blood donor screening for antibodies to Trypanosoma cruzi was revived in 2007 as part of measures to ensure the safety of the blood supply. The process combines the application of questionnaires to assess risk factors, such as travel history or residence in endemic areas, with external serological testing for high-risk donors [41]. Methods such as chemiluminescent and enzyme-linked immunosorbent assay are widely used, complemented by advanced technologies such as indirect immunofluorescence and nucleic acid testing. Donors with positive results are duly notified, permanently excluded from the donation system, and have their products discarded to avoid transmission risks [20,27]. In cases of positive screening for antibodies to *Trypanosoma cruzi*, mandatory additional diagnostic tests are recommended. The diagnosis of chronic Chagas disease is based on positive results in at least two different serological tests, using specific antigenic techniques and guidelines, since no single test has sufficient sensitivity and specificity to confirm infection [10]. Among the most common methods are enzyme-linked immunosorbent assay with recombinant antigens, immunoblot using secreted–excreted trypomastigote antigens, and indirect immunofluorescence [42,43]. In cases of discrepant results, such as patients with a positive result in the Wiener recombinant antigen enzyme immunoassay test and a negative result in secreted–excreted trypomastigote antigens, the diagnostic algorithm adopted by the Centers for Disease Control and Prevention includes performing a third test to resolve the discordance. This procedure ensures greater diagnostic accuracy, minimizing the occurrence of false positives or false negatives, and guarantees appropriate patient management and blood supply safety [43].

In Canada, the United Kingdom, and Australia, screening for Trypanosoma cruzi infection in blood donors is based on epidemiological questionnaires and, in some cases, educational strategies [27]. In Canada, screening involves epidemiological questionnaires and selected serological tests for high-risk donors, complemented by polymerase chain reaction assays with Trypanosoma cruzi-specific primers [44]. In the United Kingdom, strategies focus on epidemiological questionnaires to identify donors with possible exposure to the parasite. The country also invests in educational campaigns, using printed materials and social media to inform about the risks associated with the disease. Although universal testing is not adopted, donors considered high risk are permanently excluded from the donation system. In Australia, screening is based solely on risk factors such as travel history and residence in endemic areas. Universal serological testing is not performed, which makes the use of epidemiological questionnaires an essential practice. In all these countries, educational actions play an important role in raising donor awareness about the risks of Chagas disease and safety measures, with an emphasis on education through printed materials, videos, and social media campaigns [27].

In France, Germany, and Spain, governments implement distinct strategies to mitigate the risk of transfusion-transmitted Trypanosoma cruzi. In France, the approach incorporates serological testing for Trypanosoma cruzi antibodies, employing highly sensitive methodologies such as enzyme-linked immunosorbent and chemiluminescent assay. In addition, pathogen inactivation/pathogen reduction methodologies, including the Intercept system, are used but cover less than 25% of blood products [27]. Germany adopts a risk-based approach centered on pre-donation screening by targeted questionnaires, which assess donors’ exposure to potential infectious agents, and also consider factors such as birth or residence in endemic regions and/or potential contact with vectors. Also, systematic testing of collected samples is conducted using serological methods to detect antibodies and nucleic acid amplification techniques [45,46]. These strategies are complemented by the continuous monitoring of epidemiological trends, allowing adjustments to screening practices as necessary. Simultaneously, donor education is emphasized to raise awareness about the importance of honesty during the screening process. In Spain, serological screening is performed mainly on donors with risk factors identified in the previously completed questionnaires [27].

## 4. Chemoprophylaxis in Blood Banks

Due to the nature of Chagas disease pathology, in asymptomatic individuals during the chronic phase, parasitemia is characteristically low and intermittent in peripheral blood, which often allows the infection to remain unblocked, even decades after initial exposure [14,47]. This condition poses a significant risk of parasite transmission through blood transfusions [48]. Given the large number of asymptomatic, non-expressing, and untreated infected individuals, transfusion transmission still stands out as one of the main routes of dissemination of *Trypanosoma cruzi*, especially in non-endemic regions. To mitigate this issue, the implementation of methods for the detection and inactivation of the parasite in blood banks became an essential component of transfusion safety protocols. In this context, Nussenszweig and colleagues were pioneers in elucidating the effects of gentian violet—a mixture of triarylmethane dyes (crystal violet, methyl violet, and brilliant green)—on *Trypanosoma cruzi* trypomastigotes, advocating its application in blood banks as a prophylactic measure against transfusion-transmitted Chagas disease [49]. This dye was introduced into blood banks across Latin America during the 1960s, following recommendations from the World Health Organization [50,51]. However, a significant limitation associated with its use was the intense purple staining it caused on the skin and mucous membranes of transfusion recipients, raising concerns regarding its acceptability [52,53].

Chemoprophylaxis against transfusion-transmitted Chagas disease was initially performed by adding gentian violet at a concentration of 1:4000 to blood suspected of being contaminated, for a period of 24 h, the necessary duration for the eradication of *Trypanosoma cruzi* [54]. This method was useful in controlling transmission in regions highly endemic for Chagas disease in the past. In this context, innovative initiatives for the implementation of new diagnostic tests in blood banks and the investigation of chemoprophylaxis strategies have become essential.

### Advanced Methodologies and Novel Compounds for Blood Bank Safety

The parasites can survive the storage conditions of blood components, becoming a challenge for transfusion safety. In this context, the efficacy of the combination of leukoreduction by filtration and treatment with riboflavin and ultraviolet light to reduce the *Trypanosoma cruzi* load in artificially contaminated whole blood units was evaluated [55]. The leukoreductive filtration was able to reduce the number of live parasites in the blood by more than 99%. However, the combination of filtration with treatment with riboflavin and ultraviolet light was even more effective, eliminating viable parasites in the samples tested. The number of circulating parasites in whole blood in chronic patients is generally low, approximately 1 parasite equivalent/mL or less [47], which makes parasite detection even more difficult, especially after blood fractionation. Furthermore, the riboflavin–ultraviolet light method has been shown to irreversibly damage the nucleic acids of pathogens, preventing their replication, without compromising blood components essential for transfusion [56]. In this context, leukoreduction emerges as an effective strategy to prevent transfusion-transmitted *Trypanosoma cruzi* infection, especially in endemic and non-endemic countries with high rates of immigration from Latin America.

The highest concentration of parasites was detected in plasma, followed by red blood cells before leukoreduction, evidencing a significant risk of transmission of *Trypanosoma cruzi* associated with non-leukoreduced red blood cells. Thus, the combined implementation of different approaches such as ultraviolet light and riboflavin use along with leukoreduction may represent a significant advance in reducing the risk of *Trypanosoma cruzi* transmission in blood banks. This approach has shown promise as a complementary alternative to measures already implemented in blood banks, since it overcomes the limitations of serological screening and other methods that may fail to detect recently infected donors or those with low parasitaemia.

In a recent study, arylimidamides demonstrated high trypanocidal activity under blood bank storage conditions, such as at 4 °C. In in vitro studies, the arylimidamides DB745 and DB766 presented a safe profile on blood cells, not causing hemolysis at concentrations of up to 96 µM [57]. Arylimidamides are a class of chemical compounds derived from classical aromatic amidines, but with minor structural modifications. They are called “reverse amidines” and differ from diamidines in that the amidine is linked to the aromatic nucleus of the molecule through a nitrogen atom, instead of being mediated by a carbon atom. This structural change gives arylimidamides differentiated physicochemical properties, improving their efficacy and safety in combating pathogens, such as protozoan parasites including *Trypanosoma cruzi* [58]. Furthermore, arylimidamides showed rapid action, almost eliminating bloodstream trypomastigotes, the form that circulates in the blood of mammalian hosts, after only two hours of in vitro incubation. Among the compounds tested, the arylimidamide DB1831 was the most promising, preventing acute infections in experimental mouse models due to arylimidamide-pre-treated trypomastigotes inoculum. Thus, arylimidamides have the potential to replace old methods, such as the use of gentian violet, which has undesirable side effects such as purple coloration of the skin and mucous membranes. Arylimidamides may represent an effective alternative for chemoprophylaxis of blood samples, contributing significantly to transfusion safety and the control of iatrogenic transmission of Chagas disease.

## 5. Conclusions and Perspectives

Transfusion transmission of *Trypanosoma cruzi* remains a significant concern, especially in non-endemic regions, where population mobility and the absence of universal serological screening make it difficult to control this route of dissemination of *Trypanosoma cruzi* [20]. Strategic advances, such as the combination of leukoreduction with riboflavin-based treatments and ultraviolet light, have demonstrated efficacy in eliminating parasites in blood components, representing a promising solution to complement traditional serological screening practices. Leukoreduction, a process that removes leukocytes from blood components, plays an important role in reducing the transmission of intracellular pathogens such as cytomegalovirus, human T-lymphotropic virus type I/II, and trypanosomatids, including *Leishmania* and *Trypanosoma cruzi* [55,59,60,61]. Furthermore, innovative compounds, such as arylimidamides, have emerged as a promising and innovative approach in non-clinical studies and deserve further analysis. These compounds exhibit high trypanocidal activity under blood bank storage conditions, with proven safety for blood cells and the ability to eliminate parasitaemia in the short term. Among the compounds tested, DB1831 showed promising results, preventing acute infections in experimental mouse models [57].

Regarding new avenues for future research, it is important to state that although blood safety interventions have proven effective at avoiding transmission of human immune deficient virus, human B and C hepatitis, and West Nile virus, the transmission of parasites by transfusion still represents a relevant challenge [62], posing significant risks to blood recipients. Even though parasitic infections such as Malaria and Chagas disease, among others, are often regionally spread, due to human mobility, they represent a current and growing challenge to other non-endemic areas. Also, infections due to *Babesia microti*, a tick-borne protozoan parasite, are increasing, with over 200 cases of transfusion-transmitted infection recorded in North America and infections steadily increasing worldwide, including in Australia, Asia, and Europe [62].

Then, technological and therapeutic advances represent a milestone in improving transfusion safety and controlling the iatrogenic transmission of parasites such as *Trypanosoma cruzi*. The implementation of innovative approaches combined with education and dissemination of information about this parasitosis can also contribute significantly to global public health, especially in a scenario of increasing population mobility and urbanization. Continued investment in research and global standardization policies are essential to ensure blood safety in all affected regions, promoting advances in the fight against blood transmission of the etiological agent of Chagas disease.
